# Infralimbic Estradiol Enhances Neuronal Excitability and Facilitates Extinction of Cocaine Seeking in Female Rats *via* a BDNF/TrkB Mechanism

**DOI:** 10.3389/fnbeh.2019.00168

**Published:** 2019-07-31

**Authors:** Hanna Yousuf, Chad W. Smies, Madalyn Hafenbreidel, Jennifer J. Tuscher, Ashley M. Fortress, Karyn M. Frick, Devin Mueller

**Affiliations:** ^1^Department of Psychology, University of Wisconsin-Milwaukee, Milwaukee, WI, United States; ^2^Department of Biological Sciences, Kent State University, Kent, OH, United States

**Keywords:** cocaine abuse, conditioned place preference (CPP), estrogens, brain-derived neurotrophic factor, electrophysiology, intrinsic excitability, extinction learning, medial prefrontal cortex (mPFC)

## Abstract

Women are more susceptible to developing cocaine dependence than men, but paradoxically, are more responsive to treatment. The potent estrogen, 17β-estradiol (E_2_), mediates these effects by augmenting cocaine seeking but also promoting extinction of cocaine seeking through E_2_’s memory-enhancing functions. Although we have previously shown that E_2_ facilitates extinction, the neuroanatomical locus of action and underlying mechanisms are unknown. Here we demonstrate that E_2_ infused directly into the infralimbic-medial prefrontal cortex (IL-mPFC), a region critical for extinction consolidation, enhances extinction of cocaine seeking in ovariectomized (OVX) female rats. Using patch-clamp electrophysiology, we show that E_2_ may facilitate extinction by potentiating intrinsic excitability of IL-mPFC neurons. Because the mnemonic effects of E_2_ are known to be regulated by brain-derived neurotrophic factor (BDNF) and its receptor, tropomyosin-related kinase B (TrkB), we examined whether BDNF/TrkB signaling was necessary for E_2_-induced enhancement of excitability and extinction. We found that E_2_-mediated increases in excitability of IL-mPFC neurons were abolished by Trk receptor blockade. Moreover, blockade of TrkB signaling impaired E_2_-facilitated extinction of cocaine seeking in OVX female rats. Thus, E_2_ enhances IL-mPFC neuronal excitability in a TrkB-dependent manner to support extinction of cocaine seeking. Our findings suggest that pharmacological enhancement of E_2_ or BDNF/TrkB signaling during extinction-based therapies would improve therapeutic outcome in cocaine-addicted women.

## Introduction

Susceptibility to developing cocaine abuse disorders is higher in females than in males, an effect mediated by the actions of estrogens (McCance-Katz et al., 1999; Elman et al., 2001; Lynch et al., 2002; O’Brien and Anthony, 2005; Lejuez et al., 2007; Becker and Hu, 2008; Evans and Foltin, 2010). Previous work has shown that the potent form of estrogen, 17β-estradiol (E_2_), promotes formation and expression of drug-related memories in females (Lynch et al., 2001; Larson et al., 2007; Evans and Foltin, 2010; Bobzean et al., 2014; Segarra et al., 2014; Doncheck et al., 2018) and enhances learning and memory across multiple behavioral paradigms (Daniel, 2006; Frick, 2012, 2015). Paradoxically, the mnemonic effects of E_2_ facilitate extinction of cocaine seeking in female rats and lack of E_2_ in ovariectomized (OVX) female rats results in extinction failure leading to perseverative cocaine seeking across more than 40 days (Twining et al., 2013). Systemic administration of E_2_, however, rescues extinction learning in these rats (Twining et al., 2013). Extinction memories to suppress fear- and drug-associated behaviors are consolidated in the infralimbic-medial prefrontal cortex (IL-mPFC; Quirk et al., 2000; Peters et al., 2008; Quirk and Mueller, 2008; Torregrossa and Taylor, 2013), but the effects of E_2_ in this region on neuronal function and extinction learning are unknown. Previous work has demonstrated that E_2_ alters dorsal hippocampus (DH) function, enhancing neuronal excitability during the proestrus (high E_2_) phase relative to the metestrus (low E_2_) phase of the rat estrous cycle (Scharfman et al., 2003). Whether E_2_ acts within IL-mPFC to promote extinction learning and alter IL-mPFC neuronal excitability remains to be determined.

E_2_ may promote learning-related plasticity and intrinsic excitability by targeting neurotrophic factors such as brain-derived neurotrophic factor (BDNF) and its high-affinity receptor, tropomyosin-related kinase B (TrkB; Singh et al., 1995; Aguirre and Baudry, 2009; Hill, 2012; Wu et al., 2013; Fortress et al., 2014; McCarthny et al., 2018; Lu et al., 2019). For example, a single subcutaneous injection or direct infusions of E_2_ into the hippocampus increase BDNF protein levels in this region (Gibbs, 1999; Fortress et al., 2014). Furthermore, bath-application of a Trk receptor antagonist, K-252a, to hippocampal slices attenuates neuronal excitability during the proestrus (high E_2_) phase of the rat estrous cycle (Scharfman et al., 2003), suggesting that E_2_ may enhance neuronal excitability *via* a BDNF/Trk-dependent mechanism. Whether the interaction between E_2_ and BDNF/Trk signaling is necessary for IL-mPFC neuronal excitability and whether this interaction supports extinction learning remains unknown.

Using a cocaine conditioned place preference (CPP) paradigm, we determined if infusions of E_2_ in IL-mPFC would facilitate extinction of cocaine seeking in OVX female rats. To assess the physiological effects of E_2_, we used patch-clamp electrophysiology to investigate if bath-application of E_2_ would enhance intrinsic excitability of IL-mPFC neurons and whether E_2_-mediated excitability could be prevented in the presence of Trk receptor antagonists. Additionally, we tested whether E_2_-BDNF interactions were necessary for extinction of a cocaine CPP in OVX female rats. Our results reveal that E_2_ enhances excitability in IL-mPFC neurons *via* BDNF/Trk signaling to promote extinction of a cocaine CPP.

## Materials and Methods

### Subjects and Surgery

Female Long-Evans rats weighing between 275 and 300 g were individually housed in clear plastic cages. Rats were maintained on a 14-h light/10-h dark cycle and had unlimited access to water and standard laboratory chow (Teklad, Harlan Laboratories). Rats were weighed and handled daily for approximately 3 days prior to surgery and before the start of experiments. All experimental protocols were approved by the Institutional Animal Care and Use Committee at the University of Wisconsin-Milwaukee in accordance with National Institute of Health guidelines.

Surgeries were performed as previously described (Frick et al., 2004; Otis et al., 2013; Twining et al., 2013). Rats were anesthetized with a mixture of ketamine/xylazine (90/10.5 mg/kg, i.p.) and underwent bilateral OVX using a dorsal approach (Frick et al., 2004). A single, horizontal incision was made along the spine and the ovary was isolated. The tip of the uterus was clamped and ligated and the ovary was removed with a scalpel. The remaining tissue was returned to the abdomen. The same procedure was repeated on the other ovary, and the incision was closed with sterile sutures and wound clips. For infusion experiments, rats were implanted with a double-barrel guide cannula aimed bilaterally at IL-mPFC (anteriorposterior, +2.8; mediolateral ±0.6, and dorsoventral, −4.4 mm relative to bregma). Following surgeries, rats were given an antibiotic (penicillin G procaine, 75,000 units in 0.25 mL) and an analgesic (carprofen, 5.0 mg in 0.1 mL) subcutaneously and then allowed to recover for approximately 10 days before behavioral testing.

### Drugs

Cocaine HCl (National Institute on Drug Abuse) was dissolved in sterile 0.9% saline at a concentration of 10 mg/mL, and administered systemically at a dose of 10 mg/kg, i.p. To ensure that E_2_ levels did not build over time from repeated infusions, a water-soluble form of E_2_ dissolved in 2-hydroxypropyl-β-cyclodextrin (HBC) that is metabolized within 24 h was used (Pitha and Pitha, 1985). HBC vehicle and HBC-encapsulated E_2_ were dissolved in sterile 0.9% saline (0.2 mg/mL) and injected i.p. at a dose of 0.2 mg/kg (Gresack and Frick, 2006) or directly infused into IL-mPFC at 5 μg/0.5 μl/side (Fernandez et al., 2008). ANA-12 (selective TrkB receptor antagonist) was dissolved in 1% DMSO in physiological saline (Zhang et al., 2015) and administered i.p. at 0.5 mg/kg (Cazorla et al., 2011). For electrophysiological recordings, 25 nM β-estradiol (not HBC encapsulated) was dissolved in 100% DMSO and diluted with artificial cerebral spinal fluid (aCSF) to a final DMSO concentration of 0.0001%. K-252a (Trk receptor antagonist) was dissolved in 100% DMSO and bath-applied at 100 nM and diluted with aCSF to a final concentration of 0.001% DMSO (Montalbano et al., 2013).

### Patch-Clamp Electrophysiology

Female rats aged 3 months were OVXed and allowed to recover for 7 days. Rats received systemic injections of E_2_ or HBC vehicle for 3 days before being euthanized for patch-clamp recordings. They were anesthetized with isofluorane, and their brains were rapidly removed and transferred to ice-cold, oxygenated (95%/O_2_/5% CO_2_) aCSF containing the following composition (in mM): 124 NaCL, 2.8 KCl, 1.25 NaH_2_PO_4_, 2 MgSO_4_, 2 CaCl_2_, 26 NaHCO_3_, and 20 dextrose. Coronal slices were cut 400 μM in ice-cold aCSF using a vibrating blade microtome (Leica VT1200). Slices recovered in warm aCSF (32–35°C) for 30 min followed by incubation in room-temperature aCSF for the remainder of the experiment. Slices were transferred to a submersion chamber, mounted, and perfused with aCSF (~2 ml/min; room temperature). Pyramidal neurons with visible apical dendrites in layer 5 of the IL-mPFC were visualized with differential interference contrast using a 60× water-immersion lens on an upright Eclipse FN1 microscope (Nikon Instruments). Whole-cell patch recordings of IL-mPFC pyramidal neurons were obtained using fire polished borosilicate glass pipettes (3–8 MΩ), filled with internal solution containing the following (in mM): 110 K-gluconate, 20 KCL, 10 HEPES, 2 MgCl_2_, 2 ATP, 0.3 GTP, 10 phosphocreatine; 0.2% biocytin, pH 7.3, and 290 mOsm. Intrinsic excitability was obtained with current clamp using a MultiClamp 700B (Molecular Devices) patch-clamp amplifier connected to a Digidata 1440A digitizer (Molecular Devices). The liquid-liquid junction potential (measured as 13 mV) was compensated for all recordings. All electrophysiological data were analyzed using Clampfit (Molecular Devices).

After 10 min of stable recordings, layer 5 IL-mPFC neurons were polarized to approximately −60 mV to control for variance in resting membrane potential. A series of 1 s current steps were applied (−40 to 500 pA; 10 pA steps) and the number of evoked action potentials (APs) was recorded to measure basic membrane properties (Table 1). To measure input resistance, current pulses were injected and the resulting voltage deflections were measured to create a V-I plot. Furthermore, a rheobase was measured, which is the minimum amount of current required to elicit a single AP. Rheobase was analyzed in a subset of layer 5 pyramidal neurons by applying 1 s current steps with 10 pA increments until a single AP was elicited. Intrinsic excitability was measured by applying a 2 s depolarizing step every 7.5 s and evoked APs were recorded. The level of depolarizing step was adjusted to rheobase and remained constant throughout the experiment (Otis et al., 2013). To measure effects of E_2_ on intrinsic excitability, 25 nM of E_2_ was bath-applied and baseline current steps were applied every 5 min for approximately 30 min. To ensure that excitability was enhanced by E_2_ and not by the current pulses on its own, the same protocols were repeated for the same amount of time in slices that remained in aCSF and did not receive any E_2_ treatment. To measure the effects of K-252a on E_2_-induced excitability, slices were bathed in 100 nM K-252a (Montalbano et al., 2013) for approximately 20 min before bath-application of E_2_. Following recording, brain slices were fixed in phosphate-buffered formalin overnight.

**Table 1:** Effects of E_2_ on intrinsic excitability of infralimbic-medial prefrontal cortex (IL-mPFC) pyramidal neurons.

Group	Time	R_N_ (MΩ)	V_Rest_ (MΩ)	Rheobase (pA)	Threshold (mV)	Amplitude (mV)	Half width (ms)	sAHP (ms)	mAHP (ms)	fAHP (ms)
Systemic HBC	Pre E_2_	272.3 ± 33.3	−58.9 ± 0.5	29.0 ± 3.8	−35.8 ± 0.6	73.5 ± 3.0	1.0 ± 0.1	0.0 ± 0.2	2.2 ± 0.5	19.0 ± 1.1
	Post E_2_	308.0 ± 39.5*	−58.5 ± 0.7	18.0 ± 3.2*	−38.3 ± 0.8**	71.5 ± 3.5	1.0 ± 0.1	0.2 ± 0.2	2.3 ± 0.5	16.1 ± 1.1*
Systemic HBC	Pre E2 + K-252a	291.7 ± 42.2	−59.7 ± 0.5	35.7 ± 4.8	−34.0 ± 0.6	67.8 ± 3.3	1.4 ± 0.1	0.2 ± 0.2	3.9 ± 0.7	22.7 ± 1.1
	Post E_2_ + K-252a	329.7 ± 41.5*	−60.7 ± 0.4	37.1 ± 5.7	−35.4 ± 0.5	64.3 ± 3.5	1.5 ± 0.1	0.4 ± 0.2	4.1 ± 0.7	22.9 ± 0.8
Systemic E_2_	Pre E_2_	282.3 ± 35.3	−59.9 ± 0.6	33.3 ± 5.8	−35.3 ± 1.0	72.5 ± 2.6	1.3 ± 0.1	0.1 ± 0.3	2.5 ± 0.8	19.6 ± 1.5
	Post E_2_	318.0 ± 45.1*	−58.5 ± 0.5	26.7 ± 6.0*	−37.2 ± 1.1**	71.4 ± 2.7	1.3 ± 0.1	0.0 ± 0.2	2.3 ± 0.4	17.6 ± 1.4

E_2_, 17β-estradiol; R_N_, input resistance; V_rest_, resting potential; sAHP, slow afterhyperpolarization; mAHP, medium afterhyperpolarization; fAHP, fast afterhyperpolarization. *p < 0.05, **p < 0.001.

To confirm that patch-clamp recordings were from layer 5 IL-mPFC neurons, biocytin-filled pyramidal neurons were washed in 0.1 M phosphate-buffered saline (PBS), followed by 1% NaBH_4_ in PBS and 10% normal goat serum. The slices were incubated overnight with 3% NGS, 0.2% Triton-X, and PBS. After 24 h, slices were washed in PBS and incubated for 2 h with a green fluorescent antibody (streptavidin, 1:250). Slices were washed with PBS before being mounted with antifade mounting medium and coverslipped and were visualized using 20× magnification with green fluorescent light, to locate neurons and verify that they were pyramidal.

Electrophysiological data were analyzed using Clampfit (Molecular Devices). Basic neuron properties were examined: input resistance, resting membrane potential, AP half-width, AP amplitude, and AP threshold (Table 1). To analyze slow afterhyperpolarization (sAHP), voltage was recorded 1 s following current offset, which was then subtracted from baseline voltage before current injection (Kaczorowski et al., 2012). To analyze medium AHP (mAHP), voltage was recorded 150 ms following current offset, which was then subtracted from baseline voltage before current injection (Song et al., 2015). Fast AHP (fAHP) was measured as the antipeak amplitude relative to AP threshold (Song and Moyer, 2017), which was then subtracted from baseline. Basic measures of intrinsic excitability were analyzed using independent samples *t*-test before and immediately following drug application. To analyze number of APs, the average number of spikes was plotted against time. Repeated-measures ANOVA was used to compare excitability across time and between groups.

### Conditioned Place Preference

Testing and conditioning were conducted in a 3-chamber apparatus in which two larger conditioning chambers (33 × 23 × 29 cm) were separated by a smaller chamber (15 × 18 × 29 cm). The larger conditioning chambers had wire mesh flooring with white walls, whereas the other had gold-grated flooring with a black wall. The center chamber had aluminum sheeting as flooring. All floors were raised 4 cm, with removable trays placed beneath. Removable partitions were used to isolate the rats within specific chambers during conditioning. During baseline and CPP trials, the doors were removed to allow free access to the entire apparatus. Each of the larger chambers contain two infrared photobeams separated by 8 cm. If the beam furthest from the door was broken, then the rat was considered to be in the larger chamber. If only the beam closest to the center chamber was broken, then the rat was considered to be in the center chamber. During all phases of the experiments, the room was kept in semi-darkness.

A pre-test determined baseline preferences by placing the rats into the center chamber with free access to the entire apparatus for 15 min and recording time in each chamber. Rats spent an equal amount of time in the larger conditioning chambers, but less time in the center chamber. ANOVA revealed an effect of chamber for all rats during baseline test (*F*_(2,204)_ = 98.73, *p* < 0.001), and *post hoc* analyses confirmed that less time was spent in the center chamber than either of the conditioning chambers (*p* < 0.001). Therefore, an unbiased procedure was used, in which rats were randomly assigned to receive cocaine in one of the two larger chambers, independent of baseline preference scores. After a pre-test, rats were conditioned to associate one chamber, but not another, with cocaine in a counterbalanced fashion over 8 days. Systemic cocaine injections were given immediately before placing the rats in their chambers for 20 min conditioning sessions. Following conditioning, rats went through extinction training in which they were placed into the center chamber and allowed free access to the entire apparatus for 15 min.

All rats received daily 0.2 mg/kg, i.p. (Gresack and Frick, 2006) injections of E_2_ throughout the conditioning phase. Rats received E_2_ treatment 1 h before eight conditioning trials. Conditioning trials consisted of four pairings with cocaine and four pairings with saline. Following conditioning, rats remained in their homecages for 2 days. To test whether infusions of E_2_ in IL-mPFC facilitated extinction, HBC vehicle and HBC-encapsulated E_2_ (0.5 μg/0.5 μl/side; Fernandez et al., 2008) were directly infused in IL-mPFC 5 min prior to each extinction trial. We examined whether inactivation of TrkB receptors impairs extinction by systemically administering the selective TrkB receptor antagonist, ANA-12. One hour prior to each extinction trial (15 min), rats received either systemic injections of 0.2 mg/kg, i.p. E_2_ (Gresack and Frick, 2006) and 0.5 mg/kg, i.p. ANA-12 (Cazorla et al., 2011; Zhang et al., 2015) or E_2_ and vehicle. ANA-12 was systemically administered 1 h prior to a CPP trial because active concentrations have been detected as early as 30 min and up to 6 h after systemic injections (Cazorla et al., 2011).

After behavioral testing, rats were euthanized with an overdose of ketamine and perfused with 0.9% saline followed by 10% phosphate-buffered formalin. Brains were removed and placed in 30% sucrose/formalin solution. Following brain submersion, 40 μM thick coronal sections were sliced using a microtome from brain regions in which cannula were implanted. Sections were then mounted and stained with cresyl violet. Injector tip locations were confirmed using a rat brain atlas (Paxinos and Watson, 2007).

Drug-seeking behavior was analyzed using a three-way ANOVA to compare time within each chamber across trials and between groups (Twining et al., 2013; Otis et al., 2014). When appropriate main interaction effects were detected, Fisher’s LSD *post hoc* tests were used to make pairwise comparisons.

## Results

### Infusions of E_2_ in IL-mPFC Facilitate Extinction of Cocaine Seeking

**Figure 1:**
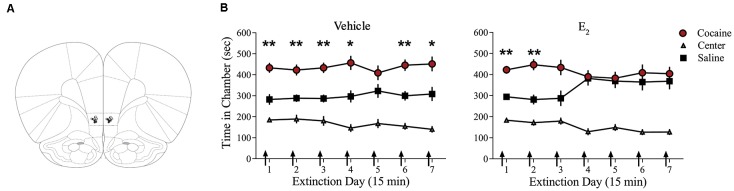
Infusions of E_2_ into infralimbic-medial prefrontal cortex (IL-mPFC) enhance extinction of a cocaine conditioned place preference (CPP). **(A)** Coronal drawings showing injector tip placements for IL-mPFC infusions. **(B)** IL-mPFC infusions (arrows) of E_2_ (*n* = 21) but not vehicle (*n* = 21) before each CPP trial promoted extinction of a CPP. **p < 0.01, and *p < 0.05. Error bars indicate SEM.

Systemic injections of E_2_ promote extinction of cocaine seeking in OVX female rats (Twining et al., 2013), but the site of action of E_2_ is unknown. Consolidation of extinction of cocaine seeking is dependent on actions within the IL-mPFC (Otis et al., 2014; Hafenbreidel et al., 2015), therefore, we tested whether localized infusions of E_2_ in this region would facilitate extinction in OVX female rats. Following conditioning, rats received IL-mPFC microinfusions of E_2_ (*n* = 21; 5 μg/0.5 μl/side) or HBC vehicle (*n* = 21) 5 min before extinction (Figure 1). ANOVA revealed no significant trial by chamber by group interaction (*F*_(12,480)_ = 1.004, *p* > 0.05). However, there was a significant effect of trial by chamber (*F*_(12,480)_ = 2.554, *p* < 0.01) and an overall effect of chamber (*F*_(2,80)_ = 64.152, *p* < 0.001). *Post* *hoc* analysis confirmed that both E_2_-treated and HBC vehicle-treated rats spent more time in the previously cocaine-paired chamber than in the saline-paired chamber during the first trial (*p* < 0.01). Whereas HBC vehicle-treated rats demonstrated a significant preference for the cocaine-paired chamber during trials 1, 2, 3, 4, 6, and 7 (*post hoc* *p* < 0.05), E_2_-treated rats did not show a significant preference for the cocaine-paired chamber after trial 2 (*post hoc* *p* > 0.05). The results suggest that E_2_ acts within IL-mPFC to enhance extinction of a cocaine CPP.

### E_2_ Potentiates IL-mPFC Pyramidal Neuron Excitability

**Figure 2:**
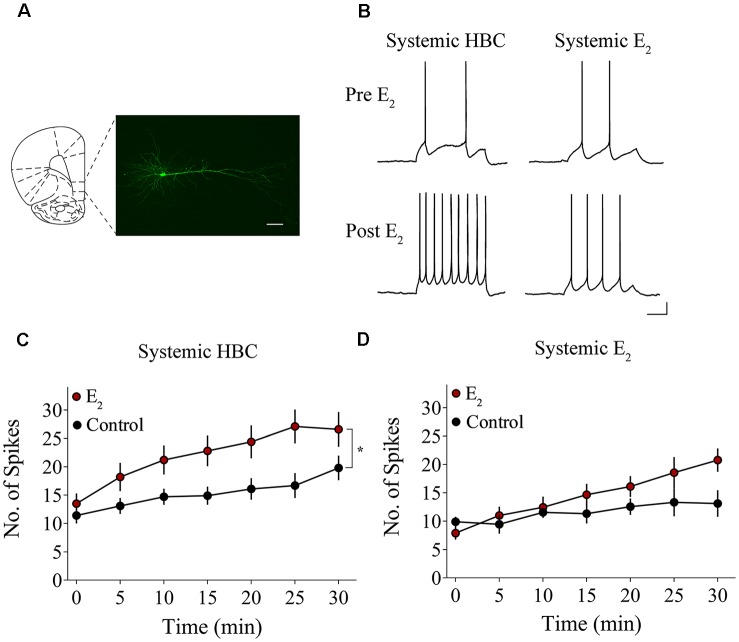
Bath-application of E_2_ potentiates IL-mPFC pyramidal neuron excitability. **(A)** Photomicrograph of a biocytin-filled IL-mPFC pyramidal neuron. Scale bar, 50 μm. **(B)** Individual traces of current-evoked action potentials (APs) from IL-mPFC pyramidal neurons before (top) and after (bottom) bath-application of E_2_ in slices from ovariectomized (OVX) female rats that were systemically injected with 2-hydroxypropyl-β-cyclodextrin (HBC)-vehicle (left) or with E_2_ (right). Scale bars, 10 mV (vertical) and 500 ms (horizontal). **(C)** Compared to artificial cerebral spinal fluid (aCSF) alone (*n* = 10), bath-application of E_2_ (*n* = 10) enhances intrinsic excitability in IL-mPFC neurons from OVX female rats that were systemically injected with HBC-vehicle prior to recordings. **(D)** Compared to aCSF alone (*n* = 9), bath-application of E_2_ (*n* = 9) does not enhance intrinsic excitability in IL-mPFC neurons from OVX female rats that were systemically injected with E_2_ prior to recordings. *p < 0.05. Error bars indicate SEM.

We tested whether bath-application of E_2_ alters intrinsic excitability in identified layer 5 IL-mPFC pyramidal neurons (Figure 2A). To control for hormonal manipulations prior to recordings, OVX female rats were systemically injected with E_2_ or HBC vehicle for 3 days before they were euthanized for patch-clamp recordings. Slices extracted from OVX female rats that were previously injected with HBC vehicle were incubated in 25 nM E_2_ or aCSF and the number of evoked APs were recorded from IL-mPFC neurons (Figures 2B,C). To ensure that excitability was drug-dependent and not due to the current injections, cells in the control group (*n* = 10) received the same number of current injections over time as cells in the E_2_ group (*n* = 10; Figure 2C). Bath-application of E_2_ (25 nM) increased the number of evoked APs in IL-mPFC neurons (Figure 2C). ANOVA revealed a significant effect of time (*F*_(6,108)_ = 28.404, *p* < 0.001) and a treatment by time interaction (*F*_(6,108)_ = 3.599, *p* < 0.05). *Post hoc* tests confirmed that bath-application of E_2_ significantly increased the number of APs compared to controls (*p* < 0.05). E_2_ did not cause membrane depolarization or reduce sAHP (a 1–2 s hyperpolarization that follows a train of APs) but significantly reduced fAHP (a 2–5 ms hyperpolarization that follows a single AP, carried by the calcium- and voltage-dependent BK channel; Storm, 1987) of IL-mPFC neurons (Table 1). Thus, bath-application of E_2_ enhances excitability of IL-mPFC pyramidal neurons from OVX female rats previously treated with HBC vehicle.

Slices extracted from OVX female rats that were previously treated with E_2_ were incubated in 25 nM E_2_ or aCSF and the number of evoked APs were recorded from layer 5 IL-mPFC neurons (Figures 2B,D). The control group (*n* = 9) received the same number of current injections over time as the E_2_ group (*n* = 9; Figure 2D). Interestingly, bath-application of E_2_ (25 nM) did not increase the number of evoked APs in slices extracted from rats that had previously received systemic injections of E_2_ (Figure 2D). ANOVA revealed a significant effect of time (*F*_(6,96)_ = 10.332, *p* < 0.001), but no significant treatment by time interaction (*F*_(6,96)_ = 2.933, *p* > 0.05). In slices from OVX female rats that received systemic injections of E_2_, bath-application of E_2_ did not enhance excitability of IL-mPFC neurons.

### Trk Receptor Blockade Prevents E_2_-Induced Potentiation of IL-mPFC Pyramidal Neuron Excitability

**Figure 3:**
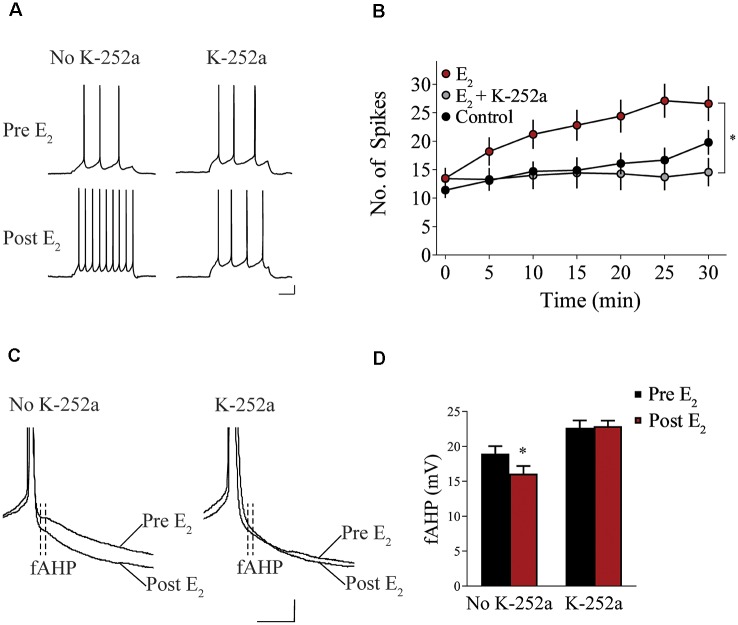
Blockade of tropomyosin-related kinase (Trk) receptors prevents E_2_-induced potentiation of IL-mPFC neuronal intrinsic excitability. **(A)** Individual traces of current-evoked APs from IL-mPFC neurons that were not incubated in K-252a (left) or were incubated in K-252a (right), before (top) and after (bottom) bath-application of E_2_. Scale bars, 10 mV (vertical) and 500 ms (horizontal). **(B)** IL-mPFC neurons treated with E_2_ (*n* = 10) have increased intrinsic excitability compared to aCSF alone (*n* = 10) or neurons that were incubated in K-252a prior to bath-application of E_2_ (*n* = 7). **(C)** Representative waveforms showing fast afterhyperpolarization (fAHP) in neurons that were not incubated in K-252a (left) or were incubated in K-252a (right), and after bath-application of E_2_. Scale bars, 10 mV (vertical) and 10 ms (horizontal). **(D)** E_2_ suppresses fAHP and this effect was blocked in the presence of Trk receptor antagonist, K-252a. *p < 0.05. Error bars indicate SEM.

We assessed whether Trk receptor blockade prevents E_2_-induced enhancement of intrinsic excitability in layer 5 IL-mPFC pyramidal neurons from OVX female rats without prior systemic E_2_ injections. Slices were first incubated for 20 min in 100 nM K-252a (Montalbano et al., 2013) and then E_2_ (25 nM) was bath-applied. The effect of E_2_ was blocked by bath-application of K-252a (Figures 3A,B), indicating that Trk receptor blockade prevents E_2_-induced potentiation of IL-mPFC neuronal excitability. E_2_ reduced fAHP and these changes did not occur when slices are incubated with K-252a (Figures 3C,D). Comparing neurons treated with E_2_ (*n* = 10), E_2_ + K-252a (*n* = 7), and aCSF alone (*n* = 10), ANOVA revealed an effect of time (*F*_(6,144)_ = 20.91, *p* < 0.001), and a treatment by time interaction (*F*_(12,144)_ = 5.546, *p* < 0.001). *Post hoc* tests confirmed that E_2_ significantly increased the number of APs compared to control or E_2_ + K-252a administration (*p* < 0.05). Together, these results show that E_2_ potentiates excitability in layer 5 IL-mPFC neurons *via* a Trk receptor-dependent mechanism.

### E_2_-Induced Facilitation of Extinction Is disrupted by TrkB Receptor Blockade

**Figure 4:**
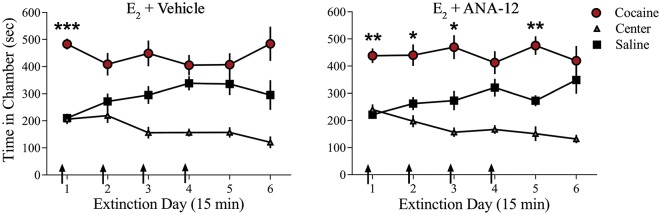
Injections of selective TrkB receptor antagonist disrupted E_2_-induced facilitation of extinction of a cocaine CPP. Systemic injections (arrows) of E_2_ + ANA-12 (*n* = 9) but not E_2_ + vehicle (*n* = 10) before the first four CPP trials impaired extinction. ***p < 0.001, **p < 0.01, and *p < 0.05. Error bars indicate SEM.

We examined whether TrkB receptor blockade prevented E_2_-facilitated extinction. Because K-252a does not cross the blood-brain barrier, we used a highly selective TrkB receptor antagonist, ANA-12, that is known to cross the blood-brain barrier effectively (Cazorla et al., 2011; Zhang et al., 2015). All rats received 0.2 mg/kg of E_2_ (Gresack and Frick, 2006) 1 h prior to each CPP test trial. In addition to E_2_ injections, rats received either a systemic injection of 0.5 mg/kg of ANA-12 (*n* = 9; Cazorla et al., 2011; Zhang et al., 2015) or vehicle (*n* = 10) 1 h before a CPP trial (Figure 4). ANOVA revealed no significant trial by chamber by group interaction (*F*_(10,170)_ = 0.1203, *p* > 0.05). However, there was a significant effect of trial by chamber (*F*_(10,170)_ = 3.926, *p* < 0.001) and an overall effect of chamber (*F*_(2,34)_ = 38.457, *p* < 0.001). *Post* *hoc* analysis confirmed that both E_2_ + ANA-12-treated and E_2_ + vehicle-treated rats spent more time in the previously cocaine-paired chamber than in the saline-paired chamber during the first trial (*p* < 0.001). However, E_2_ + vehicle-treated rats did not show a significant preference for the cocaine-paired chamber during subsequent trials (*post hoc* *p* > 0.05). In contrast, E_2_ + ANA-12-treated rats showed a significant preference for the cocaine-paired chamber during trials 2, 3, and 5 (*post hoc* *p* < 0.05). Therefore, E_2_-induced facilitation of extinction is impaired by TrkB receptor blockade.

## Discussion

Systemic E_2_ administration facilitates extinction of cocaine seeking (Twining et al., 2013), but the locus and mechanism of action have been elusive. Here we show that E_2_ acts locally within IL-mPFC to faciliate extinction of a cocaine CPP through a BDNF/TrkB-dependent mechanism. First, we found that OVX female rats fail to extinguish in the absence of E_2_ and that direct bilateral infusions of E_2_ into IL-mPFC permit extinction of cocaine seeking. Second, we show that E_2_ acts by enhancing intrinsic excitability in layer 5 IL-mPFC pyramidal neurons from OVX female rats, an effect that is blocked in the presence of a Trk inhibitor. Third, we demonstrate that E_2_-induced extinction of cocaine seeking is impaired by concurrent blockade of TrkB signaling. Thus, E_2_ interacts with BDNF/TrkB signaling in IL-mPFC to enhance neuronal excitability and facilitate extinction of cocaine seeking in female rats.

Our findings are consistent with evidence that E_2_ is necessary for extinction of both conditioned fear (Milad et al., 2010; Zeidan et al., 2011; Graham and Milad, 2013; Graham and Scott, 2018) and cocaine seeking (Twining et al., 2013). Extinction training results in the formation of a new inhibitory memory that masks the original fear or drug memory. Therefore, E_2_ could promote extinction of fear or cocaine seeking by enhancing acquisition, retrieval, and/or consolidation of the new extinction memory (Torregrossa and Taylor, 2013), the latter of which is mediated by IL-mPFC (Quirk et al., 2000; Quirk and Mueller, 2008). Previous studies demonstrated that women using hormonal contraceptives, which reduce circulating E_2_ (Rivera et al., 1999), exhibited poorer extinction recall compared to naturally cycling women (Graham and Milad, 2013). Extinction impairment was also observed in female rats treated with hormonal contraceptives, however, these impairments were restored either by exogenous treatment of E_2_ or by terminating use of hormonal contraceptive after fear conditioning (Graham and Milad, 2013). Similarly, systemic administration of E_2_ to OVX female rats promoted extinction of cocaine seeking as compared to vehicle-treated rats (Twining et al., 2013). In that study, E_2_-treated rats extinguished within a week whereas vehicle-treated rats continued to perseverate for over 6 weeks. Importantly, the extinction impairment observed in vehicle-treated OVX rats was reversed by administration of E_2_ (Twining et al., 2013). We now show that E_2_ acts locally within IL-mPFC to facilitate extinction of cocaine seeking in OVX female rats. Whether these findings extend to extinction of drug seeking across drug classes, or to extinction of natural reward seeking, remains to be tested.

In addition to showing that E_2_ acts within the IL-mPFC to facilitate extinction learning, our results are the first to reveal that E_2_ enhances intrinsic excitability in IL-mPFC neurons. Greater intrinsic excitability lowers the threshold for synaptic changes and is a neural correlate of extinction learning (Daoudal and Debanne, 2003; Santini et al., 2008; Mozzachiodi and Byrne, 2010; Sehgal et al., 2013). We found that bath-application of E_2_ increased excitability in IL-mPFC neurons from OVX female rats that did not receive systemic E_2_ injections prior to recordings. Previously, bath-application of E_2_ was shown to increase excitability in hippocampal pyramidal neurons (Wong and Moss, 1991; Kumar and Foster, 2002; Carrer et al., 2003; Woolley, 2007; Wu et al., 2011). E_2_ enhances excitability by regulating the slow Ca^2+^-activated K^+^ current (s*I*_AHP_) and suppressing sAHP in hippocampal slices from OVX female rats (Kumar and Foster, 2002; Carrer et al., 2003; Wu et al., 2011). In IL-mPFC neurons, however, E_2_ did not alter sAHP but reduced fAHP. One reason for this may be that sAHP and fAHP are differentially modulated depending on the type of learning. For example, changes in sAHP occur following fear conditioning (Santini et al., 2008), eye-blink conditioning (Moyer et al., 1996; Thompson et al., 1996), and Morris water maze learning (Oh et al., 2003), whereas fAHP (but not sAHP) is reduced after extinction learning (Santini et al., 2008). Thus, both extinction and E_2_ may enhance excitability of IL-mPFC neurons through suppression of fAHP.

Bath-application of E_2_ did not induce excitability or reduce fAHP in IL-mPFC neurons from OVX female rats that received systemic injections of E_2_ prior to recordings. These results have also been observed in hippocampal neurons where pretreatment of E_2_ prevented the effects of bath-applied E_2_ on excitability (Carrer et al., 2003). The lack of excitability changes may be due to a negative feedback mechanism as E_2_ has been shown to facilitate seizures (Newmark and Penry, 1980; Woolley, 2000). Systemic injections of E_2_ could have downregulated ERs in IL-mPFC and reduced the effects of E_2_ bath-application to prevent the induction of seizures. Future work is needed to elucidate exactly how pretreatment with E_2_ influences the extinction circuitry.

Our findings show that E_2_ potentiates IL-mPFC neuronal excitability *via* Trk receptor activation. Behaviorally, E_2_ may aid extinction of cocaine seeking by regulating BDNF/Trk signaling. BDNF is a likely target as it has been shown to promote extinction of cocaine seeking and fear learning (Peters et al., 2010; Otis et al., 2014), whereas the TrkB receptor antagonist, ANA-12, impaired extinction of cocaine seeking in male rats (Otis et al., 2014). Similarly, we demonstrate that ANA-12 prevents the facilitating effects of E_2_ on extinction of a cocaine CPP in OVX female rats. Our data support previous work that E_2_-BDNF interactions may be necessary for learning-related plasticity. For example, E_2_ replacement in young adult OVX female rats enhances BDNF protein levels in the olfactory bulbs (Jezierski and Sohrabji, 2000, 2001), hippocampus (Gibbs, 1998; Allen and McCarson, 2005; Fortress et al., 2014), cortex (Sohrabji et al., 1995; Allen and McCarson, 2005) amygdala (Liu et al., 2001; Zhou et al., 2005), septum (Gibbs, 1999; Liu et al., 2001), dorsolateral area of the bed nucleus terminalis, and the lateral habenular nucleus (Gibbs, 1999). E_2_ has also shown to increase BDNF expression in the entorhinal cortex of aged OVX female rats (Bimonte-Nelson et al., 2004) as well as in the hippocampus of gonadectomized male mice (Solum and Handa, 2002). Moreover, BDNF is necessary for E_2_ regulation of dendritic spines and ultimately synaptic transmission. E_2_-mediated increases in dendritic spine density are attenuated by inhibiting TrkB receptors with K-252a in hippocampal slice cultures (Sato et al., 2007). Furthermore, aromatase knockout mice, which have depleted neuron-derived E_2_, had a large decrease in both BDNF protein as well as dendritic spine density and these deficits were rescued by treatment with estradiol benzoate (Sasahara et al., 2007; Lu et al., 2019). Although E_2_ influences multiple brain structures to enhance cognitive processes, evidence to date indicates that E_2_ primarily targets IL-mPFC to promote extinction of fear- and drug-associated memories. For example, intrahippocampal infusions of E_2_ did not facilitate extinction of a cocaine CPP in OVX female rats (preliminary data, not shown). Moreover, systemic administration of E_2_ to naturally cycling rats enhanced c-fos activity in IL-mPFC relative to prelimbic mPFC and central amygdala during fear extinction recall (Maeng et al., 2017). Thus, E_2_ and BDNF may act synergistically to potentiate plasticity within the IL-mPFC and strengthen extinction learning.

E_2_ is known to modulate BDNF through regulation of the gene encoding BDNF as this gene contains a sequence similar to the estrogen response element (ERE; Sohrabji et al., 1995). Through the classical genomic mechanism, estrogens bind to intracellular ERs to form complexes, which bind to ERE in the DNA to influence gene transcription. ER-ligand complexes bind to the ERE-like motif on the BDNF gene and may regulate BDNF expression (Sohrabji et al., 1995). Moreover, both E_2_ and BDNF share common signal transduction pathways and transcription factors. These include signaling through the extracellular regulated protein kinase (ERK; Toran-Allerand et al., 1999; Yamada and Nabeshima, 2003; Boulware et al., 2013; Lu et al., 2019) the phosphatidylinositol 3-kinase (PI3-K; Mizuno et al., 2003; Znamensky et al., 2003; Fortress et al., 2013), Ca^2+^/Calmodulin-dependent protein kinase II (CaMKII; Sawai et al., 2002; Blanquet et al., 2003) and cAMP response element-binding protein (CREB; Ernfors and Bramham, 2003; McEwen et al., 2001; Boulware et al., 2005; Lu et al., 2019). Although there are indirect findings that E_2_ and BDNF interact to enhance memory, much more work is required to understand how E_2_ activates specific signaling pathways to regulate BDNF/Trk expression.

In conclusion, our data provide the first evidence that E_2_ localized within IL-mPFC facilitates extinction in female rats through a BDNF/TrkB-dependent mechanism. These findings have implications for the treatment of cocaine abuse in women. Cocaine dependence has increased among adolescent women between the ages of 12–17, and more women are admitted for cocaine abuse treatment compared to their male counterparts (Lejuez et al., 2007). So far, little progress has been made towards sex-specific therapeutic approaches for cocaine addiction. Treatment options, such as extinction-based exposure therapy, have had limited success without any pharmacological adjuncts (Conklin and Tiffany, 2002). Thus, pharmacological enhancement of E_2_ or BDNF/TrkB signaling may prove to be clinically relevant for the treatment of disorders in women involving maladaptive memories and behavioral inflexibility such as addiction or posttraumatic stress disorder.

## Data Availability

The datasets generated for this study are available on request to the corresponding author.

## Ethics Statement

All experimental protocols were approved by the Institutional Animal Care and Use Committee at the University of Wisconsin-Milwaukee in accordance with National Institute of Health guidelines.

## Author Contributions

HY, KF and DM designed experiments. HY and DM analyzed the data and wrote the manuscript. HY, CS, MH, JT, AF and DM carried out the experiments.

## Conflict of Interest Statement

The authors declare that the research was conducted in the absence of any commercial or financial relationships that could be construed as a potential conflict of interest.
